# Effect of protraction facemask on the temporomandibular joint: a systematic review

**DOI:** 10.1186/s12903-018-0503-9

**Published:** 2018-03-12

**Authors:** Xinqi Huang, Xiao Cen, Jun Liu

**Affiliations:** 1 State Key Laboratory of Oral Diseases, National Clinical Research Center for Oral Diseases, Department of Orthodontics, West China Hospital of Stomatology, Sichuan University, Chengdu, China; 2 State Key Laboratory of Oral Diseases, National Clinical Research Center for Oral Diseases, Department of Oral and Maxillofacial Surgery, West China Hospital of Stomatology, Sichuan University, Chengdu, China

**Keywords:** Protraction facemask, Temporomandibular joint, Orthopaedic treatment, Systematic review

## Abstract

**Background:**

The aim of this study was to assess the influence of protraction facemask (PFM) on temporomandibular joint (TMJ) of skeletal Class III malocclusion patients.

**Method:**

Literature searches were carried out electronically in five English and three Chinese databases (Cochrane Database of Systematic Reviews, the Cochrane Central Register of Controlled Trials, PubMed, Embase, MEDLINE (via Ovid), Chinese Biomedical Literature Database, China National Knowledge Infrastructure, and VIP Database). The date of the most recent search was 22 March 2017. Randomized controlled trials, controlled clinical trials, cohort studies, and before-after studies comparing the effect of PFM and other treatments on TMJ were included. The data were collected and extracted by three authors. The risk of bias in the RCTs was assessed in accordance with the Cochrane Handbook for Systematic Reviews of Interventions. For the nonrandomized studies, the risk of bias was judged with Newcastle-Ottawa Scale.

**Results:**

For the 261 articles identified, 13 studies with 522 participants were included for the final qualitative analysis. Three studies were graded as high value of evidence, while seven studies and the other three studies were graded as moderate value and low value respectively. According to the available evidence, PFM contributed to the significant increase of CondAx-SBL and the significant decrease of CondAx-ML. Thin-plate spline (TPS) analysis showed a horizontal compression in condyles. Condyles tended to move superiorly and posteriorly. Concerning the occurrence of temporomandibular disorders (TMD), PFM was not involved in aggravating TMJ symptoms and signs.

**Conclusions:**

Clinical evidence suggests that PFM might contribute to the morphologic adaptation of TMJs and displacement of condyles, and PFM may well be not a risk factor for the development of TMD.

**Electronic supplementary material:**

The online version of this article (10.1186/s12903-018-0503-9) contains supplementary material, which is available to authorized users.

## Background

The prevalence of Class III malocclusion is 23% in Asians and 3–8% in Europeans [1–7]. Class III malocclusion normally consists of deficient maxilla and/or prognathic mandible, and deficient maxilla accounts for 42–63% [8]. Protraction facemask (PFM) was invented as a type of functional appliances for adolescents with Class III malocclusion [9] and previous studies indicated that PFM could reduce the need for orthognathic surgery [10].

PFM usually consists of a forehead support, a metal frame, and a chincup as an anchorage unit [11]. As the chin serves as the anchorage region in this kind of device, a clockwise rotation force is applied directly to the mandible, causing it to be displaced downward and backward during treatment and resulting in an increased mandibular plane angle [12]. The anterior rotation of the maxilla might also contribute to this phenomenon [12, 13]. Risks of this therapy consist in posterior displacement of the condyle (which was suggested to compress the nerve and vessel mesh in the bilaminar zone) and anterior displacement of the articular disc, which could lead to clinical signs of TMD [14, 15], whereas this issue has not been unequivocally defined [16–18].

In addition, the force applied to chin was mainly (approximately 75%) transmitted to bilateral TMJs [19, 20]. It is known that TMJ condylar cartilage has sufficient stiffness for physiological shear loading and thus could bear certain force generated by normal mandibular motions [21]. However, consistent shear strain could result in fatigue, damage, deformation, and other secondary tissue damage in the articular cartilage [22–25], and then cause the degradation of the TMJs cartilage and internal derangement of TMJs [21]. However, some studies found no evidence about the force at the chin point causing TMJ problems during PFM treatment [26].

The force exerted on the chincup has been suggested to redirect the mandibular downward and backward growth, and even retard the growth of a prognathic mandible [27, 28]. TMJs are the growth center of the mandibles. Therefore, PFM treatment might contribute to the skeletal changes of TMJs. A three dimension assessment study showed that PFM lead to bone apposition at the anterior eminence of the TMJ and bone resorption at the posterior wall of the articular eminence, which correlated well with the posterior displacement of the condyle [29, 30]. Meanwhile, continuous intermaxillary traction influenced the mandibular shape [30].

Most systematic reviews and meta-analysis of PFM treatment mainly focused on its efficacy on Class III malocclusion, including the skeletal and dental changes [12, 13, 27–29, 31–34]. Until now, no systematic review is available concerning the effect of PFM on TMJs, which has been a much debated issue among clinicians in the field of orthodontics.

In this systematic review, we searched the literatures, evaluated the methodological quality of clinical trials, and summarized the evidence to elucidate the effects of PFM on the morphological change of TMJs, displacement of condyle, as well as the occurrence of TMD.

## Methods

### Protocol and registration

This systematic review protocol was registered under the PROSPERO register with the number CRD42017055343 (https://www.crd.york.ac.uk/PROSPERO/).

### Eligibility criteria

Only full-length articles, which fulfilled following criteria according to PICOS schema, were considered for inclusion in this systematic review.

1. Patients (P): all participants with any age, who were diagnosed as having skeletal Class III malocclusion.
2. The intervention group (I): PFM with or without maxillary expansion.
3. The control group (C): no treatment.
4. Types of outcome measures (O): morphologic adaptation of TMJ, displacement of mandibular condyle and disk, and occurrence of TMD signs and symptoms.
5. Study type (S): prospective and retrospective studies including randomized controlled trials (RCTs), controlled clinical trials, cohort studies, and before-after studies.

### Exclusion criteria

1. Cleft lip and palate and/or craniofacial syndrome.
2. Intervention with any other appliances.
3. Outcome without data on TMJ.

### Information sources and search strategy

Two review authors (X.H. and X.C.) conducted the electronic searches independently, and any disagreements were solved by discussion or judged by the third reviewer (J.L.).

Five English and three Chinese databases (Cochrane Database of Systematic Reviews, the Cochrane Central Register of Controlled Trials, PubMed, Embase, MEDLINE (via Ovid), Chinese Biomedical Literature Database, China National Knowledge Infrastructure, and VIP Database) were searched to 22 March 2017, with no language restrictions. We also searched six grey literature databases (EOS abstract index, IADR abstract index, clinicaltrials.gov, ISRCTN registry, Grey Literature Report, and Open Grey) and evaluated studies that were cited in the reference lists of the included articles to ensure the inclusion of all relevant studies. Details of the MEDLINE search is described in Additional file (Additional file 1: Table S1).

### Selection of studies

Two of the review authors (X.H. and X.C.) examined the titles and abstracts of the identified records and removed obviously irrelevant ones, independently and in duplicate. Any disagreement was solved by discussion or judged by a third author (J.L.).

Two review authors (X.H. and X.C.) examined full text reports of potentially eligible studies with the eligibility criteria, independently and in duplicate. Any disagreement was solved by discussion or judged by a third author (J.L.). If additional information was required, these two review authors would contact the corresponding author of the study and the study would be categorized as awaiting assessment.

The systematic review is a review of studies (not reports). As each study may have been reported in several articles, abstracts, or other reports, a comprehensive search for studies may identify many different reports from potentially relevant studies.

The criteria for comparing reports [35] were as follows:

1. Same author names;
2. Same location and setting;
3. Same specific details of the interventions (e.g. force, angle, duration);
4. Same numbers of participants and baseline data; and
5. Same date and duration of the study (which can also clarify whether different sample sizes were due to different periods of recruitment).

### Data extraction

Result data regarding participants information, intervention, follow-up periods, outcome measurements were extracted and recorded independently and in duplicate by two review authors (X.H. and X.C.). Any disagreement was solved by discussion or judged by a third author (J.L.).

### Data synthesis

We tested statistical heterogeneity by applying the chi square and I^2^ tests [36]. A low *P* value provides evidence of heterogeneity of PFM effects. And I^2^ describes the percentage of the variability in effect estimates that is due to heterogeneity rather than sampling error. If heterogeneity was high (I^2^>50%), the random-effects model was chosen for the meta-analysis; Otherwise, the fixed-effects model was adopted [37]. When there is inconsistency in the direction of PFM effect, it might be irrational to conduct a meta-analysis.

We planned to perform a meta-analysis if the data were similar enough. The measurements of PFM effect for binary data were to be expressed as relative risks along with 95% confidence intervals (CIs); as for continuous data, mean difference and 95% CIs would be used. The statistical significance of the hypothesis test was set at α = 0.05 (two tailed z tests) [37].

### Quality assessment

Two review authors (X.H. and X.C.) evaluated the risk of bias of the included studies by the Cochrane Handbook for Systematic Reviews of Interventions [38], independently and in duplicate.

For the nonrandomized studies, risk of bias was judged with the Newcastle-Ottawa Scale [39]. Using “star system”, a study usually can be awarded one star for each numbered item when it meets certain criteria. In order to give more detailed quality assessment, we regarded “to give stars” as “low risk of bias”, while regard no description as “unclear risk of bias” and the other conditions as “high risk of bias”. (Table 1).

**Table 1:** Items and criteria for quality assessment with the Newcastle-Ottawa scale

	Items	When to give stars (low risk of bias)
Selection	Representativeness of the exposed cohort	Truly or somewhat representative of the average in the community
	Selection of the control group	Drawn from the same community as the exposed cohort
	Ascertainment of the treatment group	Secure record or structured interview
	Demonstration that outcome of interest was not present at start of study	Yes
Comparability	Comparability of participants on the basis of the design or analysis	Study controls for the most important factor or any additional factor
Outcome	Assessment of outcome	Independent blind assessment or record linkage
	Was follow-up long enough for outcomes to occur?	Yes
	Adequacy of follow-up	Complete follow-up, or Subjects lost to follow-up unlikely to introduce bias, or small number lost follow-up, or description provided of those lost

For the randomized studies, risk of bias was judged from six separate domains:

1. Random sequence generation
2. Allocation concealment
3. Blinding of participants and personnel
4. Blinding of outcome assessment
5. Incomplete outcome data
6. Selective reporting

Each study was graded with A, B, or C, according to the GRADE quality analysis criteria [40]:

1. Grade A (high value): randomized clinical study or a prospective study with a well-defined control group; and clear definition of diagnosis and endpoints; and description of diagnostic reliability tests and reproducibility tests; and blinding of outcome assessment.
2. Grade B (moderate value): cohort study or retrospective study with a well-defined control group; and clear definition of diagnosis and endpoints; and description of diagnostic reliability tests and reproducibility tests;
3. Grade C (low value): Large attrition; and/or unclear definition of diagnosis and endpoints; and/or ill-defined patient material.

## Results

### Description of study characteristics

**Fig. 1:**
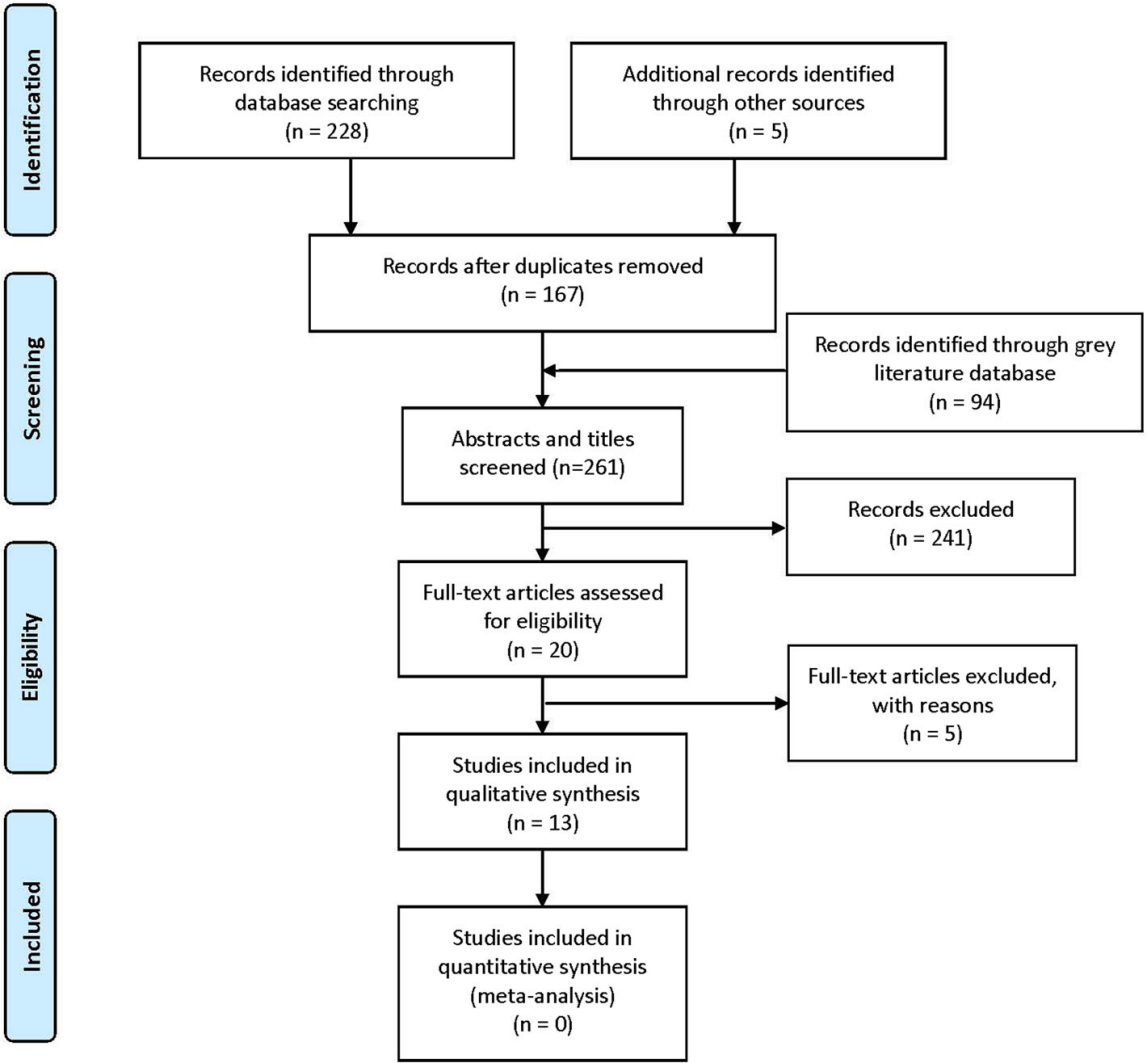
The flow chart of study selection (PRISMA flowchart)

Figure 1 shows the flow diagram. A total of 261 citations were identified from the electronic and manual search. Twenty studies were considered eligible, and full texts were retrieved after screening the titles and abstracts. Subsequently, five studies were excluded for specialized reasons [41–45] (Additional file 2: Table S2). One did not set a control group [41], one used bone-anchored maxillary protraction as intervention [42], and three did not report data on TMJ [43–45]. According to the criteria for comparing reports, we identified that two reports by Mandall (Mandall 2010 [26] and Mandall 2012 [46]) were the same study with different follow-ups, and two reports by Baccetti and Franchi (Baccetti [47] and Franchi [48]) were the same study with different measurements (cephalometric analysis and shape-coordinate Analysis, respectively). Therefore, thirteen studies [26, 46–59] were finally included in the qualitative synthesis. Two of the included studies were in Chinese and had been translated in English.

Table 2 gives an overview of the experimental setup of the included studies. All the participants involved were patients with skeletal Class III and Angle Class III malocclusion. Three studies [47, 48, 50, 51] limited patients to European-American ancestry. All the study applied PFM with or without rapid maxillary expansion, there was an exception that the study [55] by Gallagher used PFM with slow maxillary expansion. The orthopedic force was between 300 g and 600 g, the direction of elastic traction was forwards and downwards (20°-30°), and participants were asked to wear PFM for more than 10 h. Diagnostic means involve cephalometric analysis, TPS analysis, computed tomograph (CT), cone beam computed tomograph (CBCT), X ray films, electromyography (EMGs), mandibular position indicator (MPI), and research diagnostic criteria for temporomandibular disorders (RDC/TMD).

**Table 2:** Characteristics of the included studies

Author	Inclusion criteria	Number	Orthopedic force	Diagnostic means	Observation period	Drop outs
Mandall (2010) and Mandall (2012)	Anterior crossbite; Skeletal class III	PFM/RME (*n* = 35); Control (*n* = 38)	400 g, 30°, 14 h/day	Cephalometric analysis; Occlusal measurement; TMJ examination	15 m	4
					3 y	10
Kurt (2010)	Skeletal class III; Angle Class III; Anterior crossbite	PFM (*n* = 17); Control (*n* = 13)	400 g, 14 h/day	RDC/TMD	6 m	0
Baccetti (1998) and Franchi (1998)	European-American ancestry; Early or late mixed dentition; Angle Class III	PFM (*n* = 46); Control (*n* = 32)	400 g, forward and downward, Full-time basis except during meals	Cephalometric Analysis	11 ± 4 m	0
Baccetti (2000)	European-American ancestry; Early or late mixed dentition; Angle Class III	PFM/RME (*n* = 29); Control (*n* = 53)	400 g, forward and downward, 14 h/day	Cephalometric Analysis	Not reported	0
Baccetti (1999)	European-American ancestry; Early or late mixed dentition; Angle Class III	PFM/RME (*n* = 23); Control (*n* = 17)	400 g, forward and downward, Full-time basis except during meals	TPS analysis	PFM/RME: 1 ± 0.41y control: 1.9 ± 1 y	0
Chang (2006)	Chinese ancestry; Maxillary deficiency	PFM (*n* = 30); Control (*n* = 30)	300-600 g, 12 h/day	TPS analysis	9.5 m	0
Franchi (2014)	Anterior cross-bite; Angle Class III	PFM/RME (*n* = 25); Control (*n* = 16)	400-500 g, 30°, 14 h/day	TPS analysis	9.3 ± 2.2 y	0
Lee (2016)	Skeletal Class III; Maxillary deficiency; Anterior crossbite; Angle Class III	PFM (*n* = 18)	450 g, 15–30°, 16 h/day	CBCT	10.8 ± 2.4 m	0
Gallagher (1998)	Skeletal class III; Anterior crossbite	PFM/SME (*n* = 22)	300-400 g, forward and downward	Cephalometric Analysis	9 m	0
EI (2010)	Angle Class III; Maxillary deficiency; No functional anterior cross-bite	PFM (*n* = 18); Control (*n* = 16)	300-350 g, 20–25°, 14-16 h/day	MPI recordings	8.06 ± 1.63 m	0
Gong (2014)	Skeletal class III	PFM/RME (*n* = 15)	500 g, 30°, 10 h/day	CT	Not reported	0
Yao (2001)	Skeletal class III; TMD	PFM (*n* = 19)	500 g, 14 h/day	Bilateral Xray films of Schuller’s position	10.3 m	0
Ngan (1997)	Skeletal class III; Anterior crossbite	PFM (*n* = 10)	380 g, 30°, 12 h/day	masticatory muscle pain; EMG activities	Not reported	0

*PFM* protraction facemask therapy, *RME* rapid maxillary expansion, *SME* slow maxillary expansion, *EMG* electromyography; *CT* computed tomograph, *TPS* Thin-plate spline, *CBCT* cone beam computed tomograph, *RDC/TMD* research diagnostic criteria for temporomandibular disorders, *MPI* mandibular position indicator; m: months, *y* years

### Quality assessment

The detail of quality assessment is outlined in Table 3. Ten studies were of prospective design, and three studies were of retrospective design. Three studies were RCTs, five studies were cohort studies, and four studies were before-after studies. In the study by Franchi and Baccetti, the allocation was not described in detail, and this study was defined as a controlled trial.

**Table 3:** Quality assessment

Author	Study design	Study type	Definitive grade
Mandall (2010) and Mandall (2012)	Prospective	Randomized controlled trial	A
Kurt (2010)	Prospective	Randomized controlled trial	A
Ngan (1997)	Prospective	Before-after study	B
Franchi (1998) and Baccetti (1998)	Prospective	Controlled trial	B
Gallagher (1998)	Prospective	Cohort study	B
EI (2010)	Prospective	Randomized controlled trial	A
Gong (2014)	Prospective	Before-after study	C
Yao (2001)	Prospective	Before-after study	C
Baccetti (1999)	Prospective	Cohort study	B
Baccetti (2000)	Prospective	Cohort study	B
Chang (2006)	Retrospective	Cohort study	B
Franchi (2014)	Retrospective	Cohort study	B
Lee (2016)	Retrospective	Before-after study	C

According to the GRADE quality analysis, the three RCTs [26, 46, 49, 56] were graded as high value of evidence, seven studies [47, 48, 50–53, 55, 59] were graded as moderate value of evidence, and three studies [54, 57, 58] were graded as low value of evidence.

**Fig. 2:**
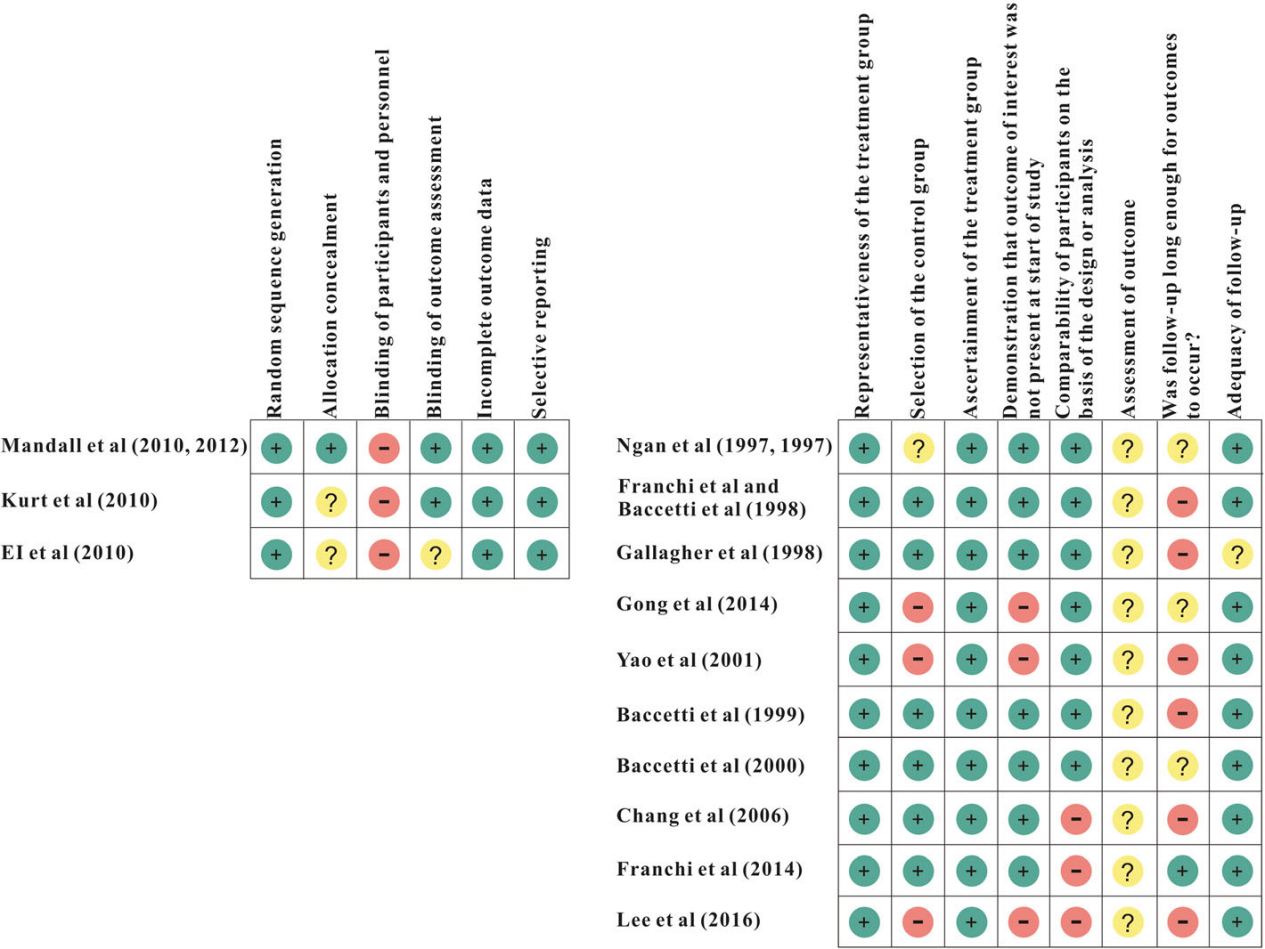
Risk of bias summary

**Fig. 3:**
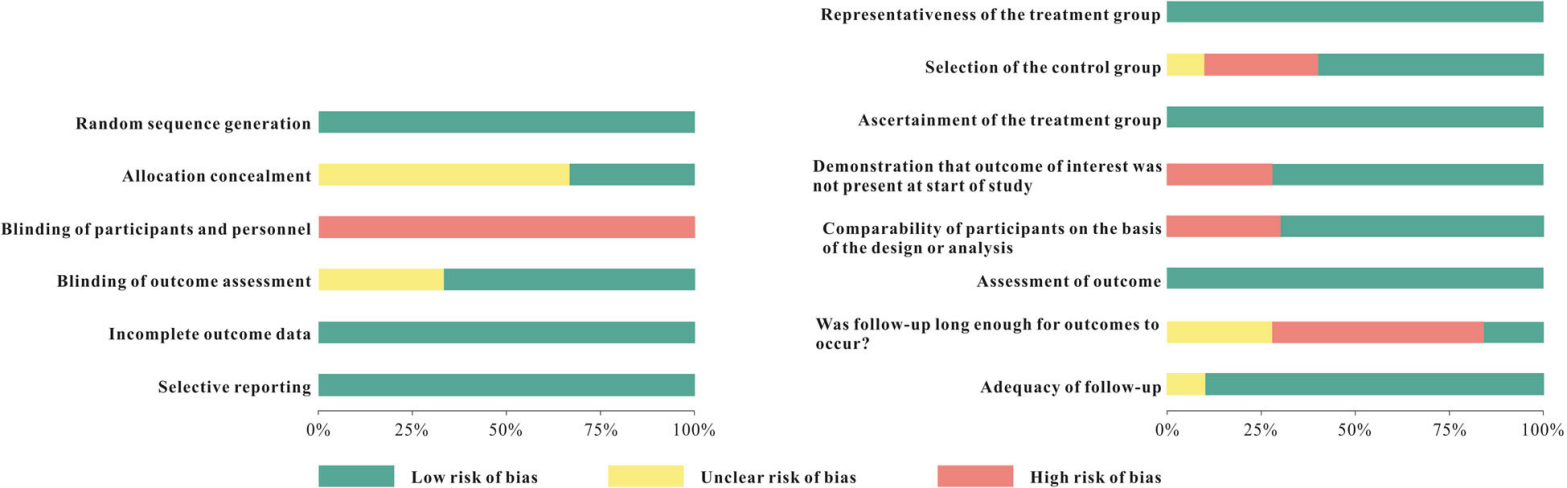
Risk of bias graph

In the three RCTs [26, 46, 49, 56], sequence generation was random. There were no incomplete outcomes data and selective reporting in these RCTs. The study by Mandall [26, 46] described allocation concealment, the studies by Kurt [49] and EI [56] did not mentioned allocation concealment (Fig. 2). In the other 10 observational studies [47, 48, 50–55, 57–59], the selection of participants was representative in the community. The ascertainment of intervention was securely recorded (Fig. 3). All the before-after studies [54, 57, 58] with no control group showed no description of the derivation of the control group, and lost comparability of participants between treatment and control groups (Fig. 2).

All the included studies lacked blinding of participants and personnel, for the PFM was evident in patients (Fig. 3).

### Description of outcomes

#### Morphologic adaptation of TMJ

Seven corresponding studies assessed the short-term outcome of morphologic adaptation of TMJ (Table 4). The interesting note was that half of them were published by Baccetti during 1998 to 2000, and his studies mainly focused on the morphologic adaptation of condyle.

**Table 4:** Morphologic adaptation of TMJ (short-term)

Author	Outcome	Short-term effect
Baccetti (1999)	TPS analysis: the shape changes of condyle	A large compression horizontally in the mandibular condyle.
Baccetti (2000)	Cephalometric Analysis: CondAx- SBL, CondAx-ML.	Significant increase of CondAx- SBL; Significant decrease of CondAx-ML; A more upward and forward direction of condylar growth significantly.
Chang (2006)	TPS analysis: the shape changes of condyle	A compression horizontallyin the mandibular condyle.
Baccetti (1998)	Cephalometric Analysis: CondAx-SBL, CondAx-ML	Significant increase of CondAx- SBL, Significant decrease of CondAx-ML; A more upward and forward direction of condylar growth significantly.
Franchi (1998)	Cephalometric Analysis: the direction of condylion movement	Significantly upward and forward movement of Condylion in direction relative to the baseline Go-Pg; Significantly downward and forward movement of Condylion in direction relative to the baseline Go-Pg.
Franchi (2014)	TPS analysis: the shape changes of condyle	A vertical extension and horizontal compression was found in the mandibular condyle.
Lee (2016)	CBCT: mandibular and glenoid fossa changes	Bone absorption at the lateral wall of the anterior mandibular fossa; Bone apposition to the medial and right anterior walls in the glenoid fossa; Bone absorption at the posterior wall and superior wall of the glenoid fossa.

*CondAx* condylar axis, *TPS* Thin-Plate Spline, *CBCT* cone beam computed tomograph

In the study by Baccetti et al. [47] and Franchi et al. [48], six of eight items showed low risk of bias (Fig. 2) and its GRADE quality was B (Table 3) which meant moderate quality. This study found that the condylion moved in a significant forward and upward direction (*P* < 0.001), the inclination of condyle to the cranial base (CondAx-SBL) showed increment significantly (8.31 mm ± 5.97 mm, *P* < 0.001), and the inclination of condyle to the mandibular line (CondAx-ML) showed decrease significantly (− 7.79 mm ± 5.94 mm, *P* < 0.001) in the early PFM-treatment group than those in the no-treatment group. Another study by Baccetti et al. [50] also showed the same results (*P* < 0.001).

TPS analysis was applied in 3 studies [51–53]. In the study by Baccetti et al. [51], six of eight items showed low risk of bias (Fig. 2) and its GRADE quality was B (Table 3). This study assessed the shape changes with TPS analysis. In the treatment group, a large compression in the horizontal axis was found in the region of condyle, while in the control group, there was a slight extension in the region of condyle. The shape changes can be interpreted as a restriction of condylar growth in sagittal direction, meaning a result of clockwise rotation of the mandible in treatment group. In the study by Chang et al. [52], five of eight items showed low risk of bias (Fig. 2) and its GRADE quality was B (Table 3). This study observed a forward compression at the condylar region near point Ar, revealing a clockwise deformation of mandible. In the study by Franchi et al. [53], six of eight items showed low risk of bias (Fig. 2) and its GRADE quality was B (Table 3). This study found a horizontal compression and vertical extension in condyle (*P* < 0.001), which was associated with a forward and upward dislocation of point Co.

The last study by Lee et al. [54] focused on the bone absorption of mandible with CBCT outcomes. In this before-after study, three of eight items showed low risk of bias and four items showed high risk (Fig. 2), and its GRADE quality was C (Table 3) which meant low quality. It was reported that in the treatment group, bone absorption was found in three-dimensional direction. In general, bone absorption of the posterior wall and bone apposition to the anterior wall in the glenoid fossa was recorded (*P* < 0.05) at the same time.

In summary, it indicates that PFM results in an upward and forward direction of condylar growth and bone remodeling in the glenoid fossa with bone apposition to the anterior wall and bone resorption of the posterior wall.

### Displacement of condyle

Six studies reported the short-term outcome on displacement of condyle (Table 5). Two included studies [26, 55] applied cephalometric analysis to this issue. The randomized controlled trial by Mandall et al. [26] showed that five of six items were low risk of bias (Fig. 2) and its GRADE quality was A (Table 3) which meant high quality. This study presented the prevalence of forward mandibular displacement on closure. Numbers of patients with a forward mandibular displacement were increased in the control group (52.6 to 70.3%), but decreased in the treatment group (52.9 to 21.9%). In the cohort study by Gallagher et al. [55], five of eight items showed low risk of bias (Fig. 2) and its GRADE quality was B (Table 3). This study found condyle moved inferiorly and posteriorly in treatment group (0.7 mm ± 1.7 mm), indicating that mandible displaced downward and backward during treatment, while no significant differences were found between treatment group and control group (*p* = 0.232).

**Table 5:** Displacement of condyle (short-term)

Author	Outcome	Short-term effect
Mandall (2010, 2012)	Cephalometric Analysis: prevalence of forward mandibular displacement on closure	70.3% have a forward mandibular displacement in control group (baseline: 52.6%); 21.9% have a forward mandibular displacement in PFM group (baseline: 52.9%).
Gallagher (1998)	Cephalometric Analysis: the direction of condylion movement	Condylion moved inferiorly and posteriorly in PFM group; No significant difference between PFM group and control group.
EI (2010)	MPI method: the direction of condyle movement, the discrepancies between the CR and MI positions	Condyle moved superiorly and posteriorly in PFM group; The discrepancies between the CR and MI positions decreased more in DFM group than in GFM group.
Gong (2014)	CT: anterior joint space, superior joint space, posterior joint space, glenoid fossa depth	No significant increment of the anterior joint space and glenoid fossa depth; Significant decrement of the superior joint space and posterior joint space.
Yao (2001)	Bilateral X-ray films of Schuller’s position: anterior joint space, superior joint space, posterior joint space, TMJ spaces area	Significant increment of the anterior joint space and the anterior joint spaces area; Significant decrement of the posterior joint space and the posterior joint spaces area; No significant increment of the superior joint space
Lee (2016)	CBCT: displacement of condyle, coronoid process, and ramus; glenoid fossa changes	Condyle showed displacement to the outside, backward, and upward; coronoid process, and ramus showed displacement to the outside.

*CT* computed tomograph, *CBCT* cone beam computed tomograph, *MPI* mandibular position indicator

The randomized controlled trial by EI et al. [56] showed that three of six items were low risk of bias and one item was high risk (Fig. 2), and its GRADE quality was A (Table 3). This study assessed the direction of condyle movement as well as the disharmony between centric relation (CR) and maximum intercuspation (MI) positions. The downward and forward movement of condyles was observed for most participators before treatment mainly because of occlusal interference caused by anterior crossbite. After treatment, condyles tended to move superiorly (− 0.97 mm ± 0.96 mm, *p* = 0.001) and posteriorly (1.13 mm ± 1.46 mm, *p* = 0.007), and meanwhile, the coincidence of CR and MI positions was achieved gradually.

The before-after study by Lee et al. [54] measured displacement of condyle by means of CBCT, and condyle showed displacement to the outside, backward, and upward in treatment group (*p* < 0.05). Meanwhile, coronoid process and ramus showed displacement to the outside (*p* < 0.05).

Two studies highlighted the quantitative changes of TMJ joint space. In the prospective before-after study by Gong et al. [57], four of eight items were low risk of bias and two items were high risk (Fig. 2), and its GRADE quality was C (Table 3). This study found that no significant differences were exhibited except posterior and superior joint space. The posterior joint space was decreased significantly from 2.56 mm ± 0.59 mm to 2.11 mm ± 0.67 mm (*p* < 0.05), and the superior joint space was also decreased from 1.69 mm ± 0.18 mm to 1.31 mm ± 0.25 mm (*p* < 0.05). The prospective before-after study by Yao et al. [58] showed that four of eight items were low risk of bias and three items were high risk (Fig. 2), and its GRADE quality was C (Table 3). This study observed the changes of anterior, superior, and posterior joint space after treatment. The anterior joint space was increased significantly from 1.48 mm ± 1.22 mm to 2.14 mm ± 1.30 mm (*p* < 0.01), and the anterior joint spaces area was increased significantly from 3.84mm^2^ ± 1.52 mm^2^ to 5.03mm^2^ ± 1.37 mm^2^ (*p* < 0.01). Meanwhile, the posterior joint space was decreased significantly from 2.59 mm ± 1.88 mm to 2.28 mm ± 1.56 mm (*p* < 0.01), and the posterior joint spaces area was decreased significantly from 6.32 mm^2^ ± 1.85 mm^2^ to 5.14 mm^2^ ± 1.29 mm^2^ (*p* < 0.01).

In summary, it suggests that PFM induced a posterior and superior movement of condyle in the short term. Eliminating any forward mandibular displacement on closure could be an important part of successful treatment, which reflects the success of PFM.

As for the long-term effect, only two studies contained related outcomes. The study by Mandall et al. [46], GRADE quality of which was A (Table 3), found that 50.0% of patients have a forward mandibular displacement in control group with the baseline of 52.6, and 21.9% have a forward mandibular displacement in PFM group with the baseline of 52.9%. The study by Gallagher et al. [55], GRADE quality of which was B (Table 3), found that condyle maintained the significantly inferior and posterior movement in PFM group (2.2 mm ± 4.7 mm, *p* < 0.05). These results were consistent with the short-term effect, affirming that the displacement effect of PFM remains stable (Table 6).

**Table 6:** Displacement of condyle (long-term)

Author	Outcome	Long-term effect
Mandall (2010, 2012)	Cephalometric Analysis: prevalence of forward mandibular displacement on closure	50.0% have a forward mandibular displacement in control group (baseline: 52.6%) 21.9% have a forward mandibular displacement in PFM group (baseline: 52.9%)
Gallagher (1998)	Cephalometric Analysis: the direction of condylion movement	Condylion moved inferiorly and posteriorly significantly.

### Occurrence of TMD

Four studies were identified for the short-term outcomes of this issue (Table 7). The study by Mandall et al. [26], whose GRADE quality was A (Table 3), measured TMJ symptoms and signs, including TMJ pain (lateral and intra-auricular), muscle tenderness (temporalis, masseter, and lateral pterygoid), crepitus, clicking, locking, and restriction of jaw movement (maximum mouth opening and lateral movement). In treatment group, lateral TMJ pain, intra-articular pain, locking, loss of movement, and temporalis/masseter spasm were not reported. 6/70 TMJs had Clicking, 2/70 TMJs had crepitus, and 1/35 patients lateral pterygoid spasm. In control group, intra-articular pain, locking, loss of movement, and temporalis/masseter spasm were not reported. 3/76 TMJs had lateral TMJ pain, 1/76 TMJs had Clicking, 9/76 TMJs had crepitus, 2/38 patients had lateral pterygoid spasm.

**Table 7:** Occurrence of TMD (short-term)

Author	Outcome	Short-term effect
Mandall (2010)	TMJ signs and symptoms	In PFM group, No patients had lateral TMJ pain, intra-articular pain, locking, loss of movement (maximum mouth opening, lateral movement), or temporalis/masseter spasm; 6/70 TMJs had Clicking; 2/70 TMJs had crepitus, 1/35 patients had lateral pterygoid spasm; In control group, 3/76 TMJs had lateral TMJ pain; no patients had intra-articular pain, locking, loss of movement (maximum mouth opening, lateral movement), or temporalis/masseter spasm; 1/76 TMJs had Clicking; 9/76 TMJs had crepitus; 2/38 patients had lateral pterygoid spasm in control group.
Kurt (2010)	TMJ signs and symptoms	In PFM group, 1/17 patients had myofascial pain; no patients had disc displacement; 3/17 patients had arthralgia; In control group, 1/13 patients had myofascial pain; no patients had disc displacement; 1/13 patients had arthralgia.
Ngan (1997)	Masticatory muscle pain	4/20 TMJs with level 1 pain of superficial masseter; 2/20 TMJs with level 1 pain of posterior temporalis; 2/20 TMJs with level 1 pain of temporal tendon; 3/20 TMJs with level 1 pain of lateral pterygoid; One month after removal of the appliance, no patients have masticatory muscle pain.
Yao (2001)	TMD	14/19 patients were TMD symptom freed; 5/19 patients were TMD symptoms relief.

The randomized controlled trial by Kurt et al. [49] showed that four of six items were low risk of bias (Fig. 2), and its GRADE quality was A (Table 3). This study utilized the Research Diagnostic Criteria for Temporomandibular Disorders (RDC/TMD) to diagnose TMD, and found that no patients had disc displacement, 1/17 patients had myofascial pain, and 3/17 patients had arthralgia after PFM. In contrast, no patients had disc displacement, 1/13 patients had myofascial pain, 1/13 patients had arthralgia in control group.

The study by Ngan et al., [59] showed that five of eight items were low risk of bias (Fig. 2), and its GRADE quality was B (Table 3). This study covered the presence of masticatory muscle pain, which is one of the major signs of TMD. All of the pain was at level 1. As a result, superficial masseter in 4/20 TMJs, posterior temporalis in 2/20 TMJs, temporal tendon in 2/20 TMJs, and lateral pterygoid in 3/20 TMJs were identified a level 1 pain in treatment group. After removal of the appliance for 1 month, no masticatory muscle pain was detected. The study by Yao et al. [58], GRADE quality of which was C (Table 3), found that 14/19 patients were freed from TMD symptoms (i.e. clicking, TMJ and muscle pain, and restriction of jaw movement) and 5/19 patients alleviated TMD symptoms.

In summary, as TMJ signs and symptoms were very low, the downwards and backwards force caused by PFM is not a risk factor of TMD in the short term.

Only Mandall et al. [46] reported the long-term outcomes. After a 3-year follow up, in treatment group, lateral TMJ pain, intra-articular pain, locking, loss of movement, or temporalis spasm was not recorded, 11% patients had Clicking, 14.1% patients had crepitus, and 3.1% patients had masseter spasm, or lateral pterygoid spasm. In control group, loss of movement or temporalis spasm was not recorded, 1.4% patients had lateral TMJ pain or intra-articular pain, 5.6% patients had clicking or lateral pterygoid spasm, 9.7% patients had crepitus, 2.8% patients had locking, or masseter spasm. From these results, TMJ signs and symptoms are still low, only crepitus tends to be increased in both groups. It is confirmed that PFM is not detrimental to TMJ in the long-term observation (Table 8).

**Table 8:** Occurrence of TMD (long-term)

Study	Outcome	Long-term effect (3y)
Mandall 2012	Percentage of TMJ signs and symptoms	In PFM group, 0% had lateral TMJ pain, intra-articular pain, locking, loss of movement (maximum mouth opening, lateral movement), or temporalis spasm; 11% had Clicking; 14.1% had crepitus; 3.1% had masseter spasm, or lateral pterygoid spasm; In control group, 1.4% had lateral TMJ pain, or intra-articular pain; 5.6% had clicking, or lateral pterygoid spasm; 9.7% had crepitus; 2.8% had locking, or masseter spasm; 0% had loss of movement (maximum mouth opening, lateral movement), or temporalis spasm.

## Discussion

The present systematic review aimed to explain three issues: firstly, whether PFM leads to morphologic adaptation of TMJ; secondly, whether PFM causes displacement of condyle; thirdly, whether PFM is responsive for the appearance of TMD.

For the first issue, two studies with moderate quality found significant increment of CondAx-SBL and significant decrease of CondAx-ML after PFM treatment, which suggested that condyle tended to grow in an upward and forward direction, and the clockwise rotation of mandible occurred in majority [47, 48, 50]. Meanwhile, TPS analysis was proposed by Bookstein in 1989 as a measuring tool for shape comparisons [60] and three included studies with moderate quality conducted it [51–53]. This analysis mainly described that horizontal compression was found in the region of condyles, which suggested the inhibition of condylar growth in sagittal plane. These short-term results yielded crucial information on the relationship between PFM therapy and bone remodeling of the condyle over a short period of time. However, as TMJs are the growth center of the mandibles, it is difficult to confirm stability of mandible morphologic adaptation without long-term studies.

As for the second issue, changes of joint space were assessed computed tomography [57] and X-ray films [58]. Significant decrement of the superior joint space and the posterior joint space supported the superior and posterior movement of condyle [57, 58]. The movement of condyle was assessed by cephalometric analysis [26, 55], MPI method [56], and CBCT [54]. Two randomized controlled trials with high quality and a before-after study with low quality indicated that the condyle moved superiorly and posteriorly [26, 54, 56]. On the contrary, the study by Gallagher et al. [55], which was a cohort study with moderate quality, reported that condyle moved inferiorly and posteriorly, but no difference between treatment and control. These two different conclusions might be due to the poor repeatability of cephalometrics analysis, whose accuracy depended on correct landmark locations on the radiographs to some degree [61]. Long-term effect of PFM on the condyle placement was consistent with the short-term effect [46, 55], indicating that the displacement effect of PFM remained stable.

The last issue is the relationship between PFM and TMD. This review found no definite evidence indicated that PFM was a risk factor in the development of TMD [26, 46, 49]. The RCT carried out by Mandall et al. [26, 46], which was high quality, found that few participants in the treatment group had the signs and symptoms of TMD, and thus refuted the hypothesis that PFM was relative with TMD. There was either no evidence that masticatory muscle pain was aggravated during PFM treatment [59]. The study by Yao et al. [58] even found that PFM would relieve the signs and symptoms of TMD. However, this study was a before-after study, which weakened the reliability of evidence. Only one of included studies covered long-term results [46], which found the prevalence of TMJ signs and symptoms were very low and almost the same with the short-term results.

A series of studies on the relationship between orthodontic treatment and TMD have been conducted for the last 3 decades [14, 62, 63]. Some studies indicated that orthodontic treatment increased the prevalence of mild signs of TMD, such as soft click and muscle tenderness on palpation [64, 65]. However, TMJ sounds without pain or functional limitation were common and most of them were normal variants, not pathologic [66, 67]. Some studies found that certain appliances (e.g. bionator and Herbst) [68–70] could alleviate the symptoms of TMD. In the present systematic review, PFM was not detrimental to TMJ and PFM could even benefit those skeletal class III malocclusion patients with TMD.

The mechanism of this process might be a correlation between TMJ adaptation and development of TMD. Condyles of most patients with skeletal class III malocclusion were located anteriorly in sagittal plane and there existed the discrepancies between the CR and MI positions [71]. PFM might contribute to the posterior movement of condyle, which could decrease the discrepancies and might relief symptoms and signs of TMJ [71]. However, present diagnosis of TMD is depending on clinical symptoms, especially on pain [72]. The assessment of pain in response to muscle palpation was only with modest, sometimes marginal, reliability, which might be because of this clinical signs are themselves unreliable, changing spontaneously over time [73]. In addition, the method of palpation (e.g. force and duration) might influence the results, and thus different examiners in different institutions might found different results from the same patient and calibration is very important [73].

Overall, the numbers of subjects enrolled in all the 13 studies were not adequate. Several studies held even fewer than 15 patients, making selection bias inevitable. The insufficiency in long-term outcomes is an obstacle to analyze the treatment effect on TMJ completely. As some studies emphasized that growth was limited, the adaptation of TMJ might temporarily correct skeletal deformity. Whether it really assists in correcting the skeletal disharmony remains unclear. Further long-term evidence is required for confirmation. Lacking the data of MRI, the included studies were deficient in the description of soft tissues such as articular disc, articular ligament, and joint capsule [74–76]. Further studies on MRI are needed in the evaluation of TMJ.

## Conclusion

This systematic review showed that current evidence supported the morphologic adaptation of TMJ (five moderate quality studies and one low quality study) and displacement of mandibular condyle (two high quality studies, one moderate quality study, and three low quality studies) caused by PFM. No evidence indicated that PFM was related with TMD (two high quality studies, one moderate quality study, and one low quality study). Further long-term and high-quality studies are needed to conclude the effect on TMJ stably. The evaluation on soft tissue is needed in future. A uniform measurement method is deeply needed to quantify the changes in TMJ caused by PFM so that conclusion can be definite and reinforced.

## Additional files

Additional file 1: Details of the MEDLINE search. (DOCX 15 kb)

Additional file 2: Reasons for exclusion of the 5 studies. (DOCX 16 kb)

## Abbreviations

CBCT: Cone beam computed tomograph
CR: Centric relation
CT: Computed tomograph
EMG: Electromyography
MI: Maximum intercuspation
MPI: Mandibular position indicator
PFM: Protraction facemask
RCTs: Randomized controlled trials
RDC/TMD: Research diagnostic criteria for temporomandibular disorders
RME: Rapid maxillary expansion
SME: Slow maxillary expansion
TPS: Thin-Plate spline
