# Supplementary material for: Effect of protraction facemask on the temporomandibular joint: a systematic review
Source: BMC Oral Health. 2018 Mar 12;18:38. doi: 10.1186/s12903-018-0503-9 (PMC5848518; doi:10.1186/s12903-018-0503-9)
Supplement: Supplementary file 1 — Details of the MEDLINE search. (DOCX 15 kb) [file 12903_2018_503_MOESM1_ESM.docx]

**S1 Table. Details of the MEDLINE search**

#1 maxillary protraction.mp. or Extraoral Traction Appliance/

#2 maxillary protractor appliance.mp.

#3 maxillary protractor.mp.

#4 reverse headgear.mp.

#5 facemask.mp.

#6 face mask.mp.

#7 Reverse Pull headgear.mp.

#8 Protraction headgear.mp.

#9 #1 OR #2 OR #3 OR #4 OR #5 OR #6 OR #7 OR #8

#10 TMJ.mp. or Temporomandibular Joint/

#11 Temporomandibular Joint.ab,ti,tw.

#12 condylar.ab,ti,tw.

#13 condyle.ab,ti,tw.

#14 Cartilage, Articular/ or Mandibular Condyle/ or condyle.mp.

#15 condyle.mp.

#16 TMJ.ab,ti,tw.

#17 #10 OR #11 OR #12 OR #13 OR #14 OR #15

#18 #9 AND #17
