# Supplementary material for: Effect of protraction facemask on the temporomandibular joint: a systematic review
Source: BMC Oral Health. 2018 Mar 12;18:38. doi: 10.1186/s12903-018-0503-9 (PMC5848518; doi:10.1186/s12903-018-0503-9)
Supplement: Supplementary file 2 — Reasons for exclusion of the 5 studies. (DOCX 16 kb) [file 12903_2018_503_MOESM2_ESM.docx]

**S2 table.** Reasons for exclusion of the 5 studies

| **Name** | | **Reason for exclusion** |
| --- | --- | --- |
| D Nardoni et al. (2015) | [55] | no control group. |
| T Baccetti et al. (2012) | [56] | The intervention with bone-anchored maxillary protraction did not match selection criteria. |
| K Kajiyama et al. (2000) | [57] | Outcome without data on TMJ. |
| C Masucci et al. (2011) | [58] | Outcome without data on TMJ. |
| P Westwood et al. (2003) | [59] | Outcome without data on TMJ. |
